# An Elusive Target: Inhibitors of JC Polyomavirus Infection and Their Development as Therapeutics for the Treatment of Progressive Multifocal Leukoencephalopathy

**DOI:** 10.3390/ijms24108580

**Published:** 2023-05-11

**Authors:** Jacob Kaiserman, Bethany A. O’Hara, Sheila A. Haley, Walter J. Atwood

**Affiliations:** Department of Molecular Biology, Cell Biology, and Biochemistry, Brown University, Providence, RI 02912, USA

**Keywords:** polyomavirus, antiviral, drug discovery, progressive multilocal encephalopathy, PML, signaling inhibitor, GW-5074

## Abstract

Progressive multifocal leukoencephalopathy (PML) is a rare demyelinating disease caused by infection with JC Polyomavirus (JCPyV). Despite the identification of the disease and isolation of the causative pathogen over fifty years ago, no antiviral treatments or prophylactic vaccines exist. Disease onset is usually associated with immunosuppression, and current treatment guidelines are limited to restoring immune function. This review summarizes the drugs and small molecules that have been shown to inhibit JCPyV infection and spread. Paying attention to historical developments in the field, we discuss key steps of the virus lifecycle and antivirals known to inhibit each event. We review current obstacles in PML drug discovery, including the difficulties associated with compound penetrance into the central nervous system. We also summarize recent findings in our laboratory regarding the potent anti-JCPyV activity of a novel compound that antagonizes the virus-induced signaling events necessary to establish a productive infection. Understanding the current panel of antiviral compounds will help center the field for future drug discovery efforts.

## 1. Introduction

Polyomaviruses are small, non-enveloped, double-stranded DNA viruses of the family *Polyomaviridae* [1,2]. Originally grouped with the papillomaviruses into the obsolete *Papovaviridae* mega-family, roughly 100 biologically and genetically distinct polyomaviruses have been identified [1,2]. Each polyomavirus is species-specific, and unique polyomaviruses have been isolated from diverse organisms, including dolphins, scorpions, monkeys, and geese [3,4,5,6]. Fourteen polyomaviruses are known to infect humans, but only four cause disease: JC polyomavirus (JCPyV), BK polyomavirus (BKPyV), Merkel cell polyomavirus, and *Trichodysplasia spinulosa*-associated polyomavirus [7,8].

Polyomavirus infection is ubiquitous, as between 60–80% of adults are seropositive for JCPyV [9,10,11,12]. JCPyV is thought to persist in the kidneys, and asymptomatic individuals secrete virus in the urine [13,14]. However, human polyomaviruses can all cause serious and often untreatable disease under immunosuppressive conditions, including those caused by lymphoproliferative disorders, HIV/AIDS, and the use of immunomodulatory drugs [7,12,13,14]. JCPyV is the causative agent of progressive multifocal leukoencephalopathy (PML), a rare but fatal demyelinating disease caused by lytic infection of oligodendrocytes and astrocytes [15]. Although PML has been known to scientists and physicians for nearly 70 years, there are no treatments for this disease [12,13,14]. 

## 2. Progressive Multifocal Leukoencephalopathy

### 2.1. History

In 1958, Åstrom, Mancall, and Richardson first described a novel demyelinating disorder characterized by a “progressive increase in the size of the lesions” in patients with chronic lymphatic leukemia and Hodgkin’s lymphoma [16]. The presence of viral inclusion bodies in oligodendrocytes and astrocytes suggested a viral origin [16]. Subsequent reports visualized virus-like particles in brain sections taken from a patient with PML, and viruses derived from this patient were cultured by 1971 [17,18,19]. These viruses were found to neither cross-react with antisera against known papillomaviruses or polyomaviruses, nor could they infect the host cells of known papillomaviruses or polyomaviruses [17,18,19]. These observations allowed for identification of a novel human virus, named JC after the initials of the patient from whom the virus was first isolated [18]. 

**Figure 1:**
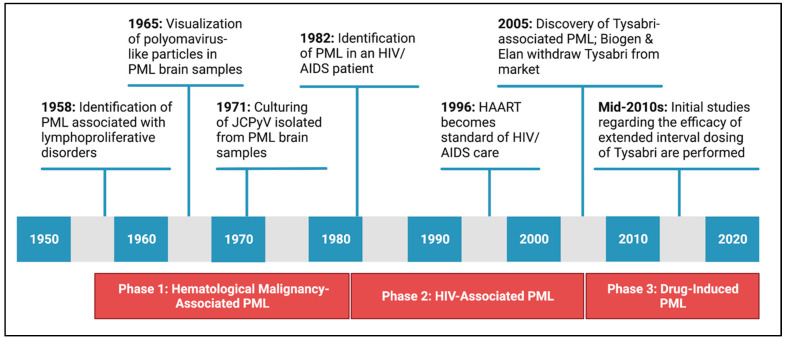
Graphical history of PML incidence. The historical incidence of PML can be separated into three distinct phases based on the primary cause of underlying immunosuppression. Created with BioRender.com.

The historical incidence of PML can be thought of in three phases (Figure 1). PML has been traditionally associated with (1) hematological malignancies, (2) HIV/AIDS, and (3) the use of immunomodulatory drugs. 

Initial cases of PML, including those published in 1958, were mostly restricted to patients with lymphoproliferative disorders. PML remained an obscure disease until the mid-1980s, as just 230 cases of PML were recorded in the United States between 1958 and 1984 [20,21]. New York City physicians identified the first case of HIV-associated PML in 1982, and during the 1980s, epidemiological studies suggest that the incidence of PML increased nearly 50-fold compared to previous decades due to the spread of HIV/AIDS [22,23,24]. PML became an AIDS-defining illness, and HIV-associated PML is estimated to have occurred in up to 10% of HIV/AIDS cases [25,26,27]. By the mid-2000s, the adoption of highly active antiretroviral therapy (HAART) significantly decreased the incidence and fatality of HIV-associated PML in the United States [23]. While HIV-associated PML is no longer the primary cause of PML, HIV-associated PML still accounts for ~80% of PML cases and causes significant morbidity [24]. 

In 2005, a new population of at-risk patients emerged when PML was associated with Biogen’s natalizumab (Tysabri), an immunosuppressive monoclonal antibody therapy [28,29,30]. Natalizumab is used to treat inflammatory conditions, including multiple sclerosis and Crohn’s disease. The risk for natalizumab-treated patients to develop PML depends on multiple factors, including prior immunosuppressant use, JCPyV-seropositivity, and length of natalizumab treatment [31,32]. Still, the incidence of natalizumab-associated PML reached 13 in 1000 for patients in the highest-risk groups, causing Biogen to recall natalizumab in 2006 [33,34]. By the mid-2010s, enhanced monitoring strategies, including extended-interval dosing schemes, decreased the incidence of PML in natalizumab-treated patients without compromising therapeutic efficacy [35,36,37]. Recent studies also conclude that a subset of PML patients (~10%) express rare deleterious variants of four genes that regulate various aspects of the immune response, including the complement cascade and antigen receptor expression on T-cells. As such, genetic screening may further reduce the incidence of PML by alerting healthcare providers to high-risk patients before beginning immunomodulatory therapies like natalizumab [38,39].

Ultimately, PML is considered a rare disease, regardless of the primary cause. As of 2023, the incidence of PML is around 1 in 200,000 [40].

### 2.2. Clinical Presentation

In all cases of PML, under immunosuppressive or immunomodulatory conditions, JCPyV invades the central nervous system (CNS) and infects oligodendrocytes and astrocytes. The timing of JCPyV migration in PML pathogenesis is controversial, as whether JCPyV enters the CNS before immunosuppression or establishes a persistent brain infection after a loss of routine immune surveillance is not known [12,13,14]. Diagnosis of PML requires (1) detection of JCPyV DNA in the cerebral spinal fluid, (2) consistent clinical presentation usually including a history of immunosuppression, and (3) characteristic white matter lesions caused by lytic infection of the myelin-producing oligodendrocytes [31]. Symptoms are varied due to the diverse localization of lesions but typically include visual deficits, motor weakness, gait disturbance, and cognitive decline [16,41,42]. Whereas the incidence of PML is rare, the prognosis of patients affected with PML is extremely poor, as PML is associated with a 30–50% mortality in the first few months after diagnosis, and patients that survive are often left with permanent neurological deficits [43,44,45,46]. 

### 2.3. Management

There are no antiviral treatments or prophylactic vaccines for PML, and treatment guidelines focus on restoring immune surveillance. Extended-interval dosing of natalizumab and HAART have reduced the incidence and severity of PML in the primary populations, but despite these successful management strategies, epidemiological, immunological, and virological obstacles still exist [31,35,36,47]. The reestablishment of immune activity is associated with immune reconstitution inflammatory syndrome (IRIS), a hyperactive immune response that occurs in up to 18% of patients with HIV-associated PML and nearly 70% of patients with natalizumab-associated PML [47,48,49]. High-dose glucocorticosteroids are prescribed to reduce PML-IRIS, but this treatment simultaneously antagonizes a patient’s ability to fight JCPyV [48,49]. In total, PML-IRIS is associated with a 28% mortality rate [47,48,49]. While the incidence of HIV- or natalizumab-associated PML has decreased, new risk groups are emerging, as reports estimate that the number of autoimmune diseases associated with PML has increased since 2006 [47]. This increase can likely be attributed to new immunomodulatory agents [50,51]. More research is therefore needed to identify viable treatment strategies for PML because there are no antiviral therapies or vaccines for this disease.

## 3. JCPyV Genome

**Figure 2:**
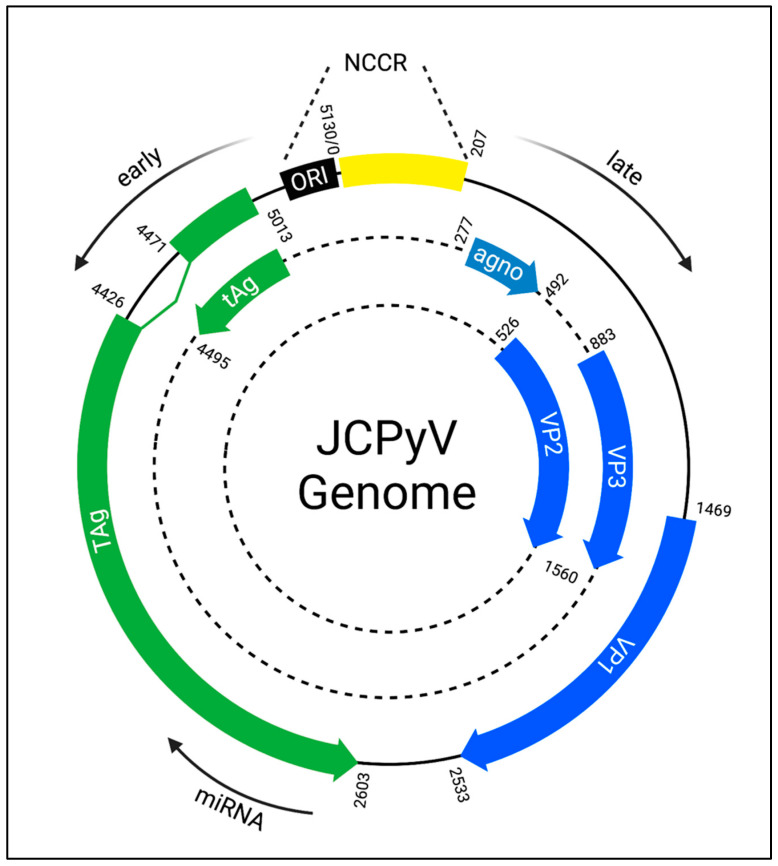
JCPyV genome. The 5130 kb JCPyV genome contains two segments: the early gene segment that includes large T-antigen (Tag) and small t-antigen (tAg), and the late gene segment that includes the structural proteins VP1, VP2, and VP3. The non-coding control region (NCCR) separates these segments, and segments are transcribed in the opposite direction. ORI = origin of replication. Agno = agnoprotein. Created with BioRender.com.

A significant body of work has characterized the genome of many polyomaviruses, including JCPyV and simian virus 40 (SV40); the SV40 genome was one of the first whole genomes synthesized [52]. Like other polyomaviruses, JCPyV contains a small, circular, double-stranded DNA genome of around 5000 base pairs (Figure 2). The JCPyV genome is separated into two segments corresponding to its biphasic replication cycle. The early gene segment, which is transcribed first, codes for large T-antigen (TAg), small t-antigen (tAg), as well as T-antigen splice variants. The late gene segment codes for the structural capsid proteins viral protein (VP) 1, VP2, and VP3, agnoprotein, and a regulatory microRNA (miR-J1) [53]. These distinct segments are separated by a non-coding control region (NCCR) containing an origin of replication, enhancers, and promoters organized into genomic blocks that are named alphabetically. Viral gene expression is regulated by complex interactions between host transcription factors, viral proteins, and the NCCR [54,55]. Transcription of each segment occurs in the opposite direction. Interestingly, while the early and late genes are organized into discrete genetic regions, the late miR-J1gene is encoded within the early TAg gene but is transcribed in the opposite sense [56]. The actions of these viral proteins as well as their potential as therapeutic targets are discussed in the following sections. 

## 4. Inhibitors of JCPyV Infection and Spread

Unlike more complex pathogenic viruses, including HIV-1 or SARS-CoV-2, the JCPyV genome only encodes one enzyme. The relative simplicity of the JCPyV genome has hindered the development of direct-acting antiviral agents, which target essential virus components. Historically, drug development efforts have disproportionately favored this approach, and direct-acting compounds make up the vast majority (~90%) of FDA-approved antivirals as of 2020 [57,58]. In contrast, the primary focus of anti-JCPyV development has been host-directed antivirals, which inhibit cellular components necessary for virus infection. Whereas host-directed antivirals may induce toxicity by purposely disrupting a host process, targeting host factors minimizes the chance a virus mutates to develop drug resistance [57]. 

**Figure 3:**
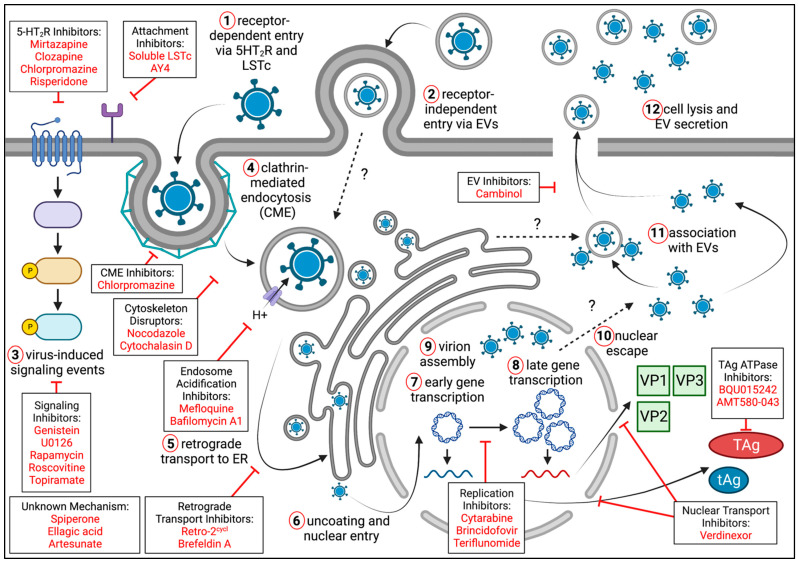
Events of the JCPyV lifecycle, including representative inhibitors of virus infection and spread. JCPyV may enter target cells as free virus through receptor-dependent mechanisms (1) and when associated with extracellular vesicles (EVs) through receptor-independent mechanisms (2). Virus-induced signaling events (3), which differ between cellular contexts, support JCPyV replication and occur immediately upon virus binding and internalization. Free virus (and potentially EV-associated virus) is internalized via clathrin-mediated endocytosis (4). JCPyV then undergoes retrograde transport to the ER (5), where virus is uncoated and released back into the cytoplasm (6). After entering the nucleus, JCPyV gene expression occurs in two discrete phases. Transcription of early phase genes (7) produces the potent oncoprotein large T-Antigen (TAg) and small t-Antigen (tAg). Late-phase gene expression (8) generates the structural proteins VP1, VP2, and VP3, which are imported back into the nucleus for virion assembly (9). JCPyV escapes the nucleus via an unknown mechanism (10) and may associate with extracellular vesicles (11). After sufficient virion production, the host cell lyses (12), releasing free virus and JCPyV-associated EVs. Representative inhibitors, including the impacted stage of the virus lifecycle, are shown in boxes. Created with BioRender.com.

Extensive research characterizing the JCPyV lifecycle, especially virus attachment, entry, and signaling, has identified many therapeutic targets for antivirals. Key steps of the virus lifecycle, as well as representative small molecule inhibitors with in vitro or in vivo anti-JCPyV activity, are shown in Figure 3 and Table 1. 

Because JCPyV causes neurological disease, evaluating whether lead compounds can accumulate in the CNS at therapeutic concentrations is crucial. 

### 4.1. Attachment Inhibitors

Viruses must enter host cells to replicate, so the initial interaction between a virus and host surface receptors plays a major role in the infectious lifecycle. Like many viruses, including coronaviruses, adenoviruses, and picornaviruses, JCPyV initially binds to sialic acids, which are monosaccharide modifications to membrane-bound lipids or proteins [59,60]. The major capsid protein of JCPyV, VP1, specifically binds to an α(2,6)-linked sialic acid moiety on lactotetrasaccharide series C (LSTc) [61,62]. This initial binding partner is distinct from the cellular receptors of other polyomaviruses, including SV40 and BKPyV, which recognize different oligosaccharides, and is critical for JCPyV infection, as mutations that disrupt LSTc binding render JCPyV non-infectious [59,61].

Decoy receptors, defined as soluble receptors that can neutralize free virus by out-competing host receptors for capsid binding sites, are an extremely promising topic in drug development. Computationally designed decoy receptors have been synthesized as a potential therapeutic against SARS-CoV-2, the causative agent of COVID-19 [63]. Soluble LSTc, but not other lactoseries tetrasaccharides, has been shown to reduce JCPyV infection [64]. Similarly, high-throughput in silico screening of ~3500 small molecules identified 11 compounds with high affinity for the JCPyV VP1-LSTc binding site. The most promising compound, AY4, inhibited JCPyV binding to SVG-A glial cells in a dose-dependent manner, as measured by flow cytometry [65]. The ability of AY4 to exert therapeutic activity within the CNS has not been investigated.

### 4.2. Entry Inhibitors

Free JCPyV has been shown to require binding to the serotonin receptor (5-HT_2_R) to infect target cells [64,66]. 5-HT_2_R, a family of three isoforms of G-protein-coupled receptors that bind the neurotransmitter serotonin, are highly expressed in the CNS and regulate diverse functions, including sleep, appetite, and mood. While 5-HT_2_R is not believed to be required for JCPyV attachment, the 5-HT_2_R-JCPyV binding event initiates a complex signaling pathway that culminates in viral entry and supports viral replication [64,66,67,68,69,70]. 

Because 5-HT_2_R is the target of many FDA-approved therapeutics for psychiatric disorders, the discovery that 5-HT_2_R mediates virus entry prompted the investigation of whether antidepressant and antipsychotic medications could be co-opted to treat PML [7]. Many 5-HT_2_R inhibitors show in vitro activity against JCPyV infection, including clozapine, ketanserin, ritanserin, metoclopramide, cyproheptadine, and mianserin [66,71,72]. While the breadth of this drug class appears promising, many 5-HT_2_R antagonists fail to slow the spread of an established JCPyV infection, which is important for managing PML because viral spread occurs long before symptom onset [71]. 

5-HT_2_R inhibitors have been used in PML patients with ambiguous results. Of these, mirtazapine has been used in single cases of idiopathic PML and drug-associated PML, and some mirtazapine-treated patients experience improvement in as early as six months following daily therapy [73,74]. Virological or clinical improvements, evaluated by JCPyV load and lesion severity, respectively, were associated with nightly mirtazapine use in four outpatient cases of HIV-associated PML, with the most significant improvement occurring when mirtazapine therapy began closest to symptom onset [75]. Additional cases of HIV-associated PML have been shown to respond to mirtazapine in combination with other antiviral agents, including mefloquine, cytarabine, cidofovir, and interferon α [76,77,78]. However, one meta-review of 5 cohort studies and 74 case reports identified little additional benefit to mirtazapine therapy [79]. Oral administration of the second-generation antipsychotic risperidone achieved complete disease remission in one case of lymphoblastic leukemia-associated PML, and a combination therapy of risperidone, mefloquine, and mirtazapine was associated with improved neurological findings in two cases of autoimmunity-associated PML and drug-associated PML [80,81,82]. While promising, these clinical data have been generated by individual case reports and more research is needed to assess the efficacy of 5-HT_2_R inhibitors in PML. 

Following attachment and 5-HT_2_R binding, JCPyV is primarily internalized via clathrin-mediated endocytosis (CME) [59,68,83]. While also a 5-HT_2_R antagonist, the second-generation antipsychotic chlorpromazine also decreases JCPyV infection by preventing clathrin recycling [83]. However, combination therapy of chlorpromazine, high-dose cidofovir, and immunoglobulins in lymphoblastic leukemia-associated PML was associated with worsening demyelination and death after 3 months of treatment [84]. Additionally, the maximum plasma and CNS concentrations of chlorpromazine are significantly lower than concentrations shown to be therapeutic in vitro, which may hinder its applicability in the clinic [7]. However, multiple compounds that antagonize different components of CME, such as Pitstop2 and Dynole 34-2, have been shown to reduce JCPyV infection in vitro, which underscores the potential for CME as an anti-JCPyV target [68]. JCPyV may also undergo macropinocytosis as an alternative internalization mechanism, as EIPA, an inhibitor of macropinocytosis, also reduced JCPyV uptake and infection in a reversible manner [85]. 

A significant complication of targeting virus attachment and entry is the association of JCPyV with extracellular vesicles (EVs), which Morris-Love et al. define as receptor-independent infection [86]. Recent research shows that JCPyV is packaged in EVs, which are small, lipid-bound vesicles secreted by infected cells [85,87,88]. This host-derived membrane shields JCPyV from neutralizing antibodies, and cells without LSTc and 5-HT_2_R can still be infected by JCPyV-associated EVs, but cannot be infected by free JCPyV [85,88]. Moreover, JCPyV-associated EVs have been shown to enter cells by clathrin-dependent and clathrin-independent mechanisms of endocytosis depending on the cell type of origin [85]. The difficulties associated with this receptor-independent infection mechanism suggest that attachment and entry inhibitors, including LSTc analogs, 5-HT_2_R inhibitors, and CME inhibitors are unlikely to reduce the infection or spread of JCPyV in vivo, as JCPyV-associated EVs appear to play an important role in PML pathogenesis [87].

### 4.3. Endosome Acidification Inhibitors

JCPyV relies on membrane-bound vesicles called endosomes for intracellular transport, and virus colocalizes with endosomal markers as early as 15 min post internalization [89]. Like other viruses, including HIV and SARS-CoV-2, acidification of these endosomes is required for JCPyV infection [7,90]. Endosome acidification inhibitors have therefore received significant attention as novel antivirals. The bacterial products bafilomycin A1 and monensin reduce JCPyV infection at nanomolar concentrations by inhibiting membrane-bound proton pumps [90,91]. A high-throughput screen identified mefloquine, a treatment for chloroquine-resistant malaria, as a potential anti-JCPyV compound due to its ability to antagonize endosomal acidification [7,92]. Because mefloquine has an established reputation as a well-tolerated, pharmacologically active compound, many studies have evaluated the therapeutic efficacy of monotherapies and combination therapies featuring mefloquine. A randomized clinical trial of mostly HIV-associated PML patients showed no significant difference associated with mefloquine monotherapy as evaluated by JCPyV loads and clinical or virological improvement [93]. Because high concentrations of mefloquine could be detected in brain samples, the lack of antiviral efficacy associated with mefloquine treatment is unlikely to be attributed to failure to cross into the CNS [93]. However, some individual case studies report reductions of JCPyV DNA and clinical or virological improvement when patients are treated with mefloquine in combination with the 5-HT_2_R antagonist mirtazapine [76,77,93,94,95,96]. In these cases, separating the effects of each drug appears difficult. More research is therefore needed to understand the clinical efficacy of mefloquine monotherapy and combination therapy, especially in PML cases not associated with HIV/AIDS. 

### 4.4. Signaling Inhibitors

While research has extensively characterized the early events in the viral lifecycle, the complex symphony of virus-induced signaling events required to support JCPyV infection is only beginning to be understood. Recent research has shown that these signaling events are highly context-dependent and differ between model cell types [70,97,98]. Additionally, how these signaling events change during receptor-independent infection via EVs is not clear. Nevertheless, because there are many already-approved cancer therapeutics that antagonize signaling cascades, the development of signaling inhibitors is a promising new direction of anti-JCPyV drug discovery. 

Many studies of JCPyV are conducted in the SVG-A fetal glial cell line, which is transformed with SV40 Large T-antigen [99]. In these cells, JCPyV activates the mitogen-activated protein kinase extracellular response kinase (MAPK-ERK) pathway [67,69,70,97,100,101]. The MAPK-ERK pathway mediates cellular responses to the external stimuli that direct cells to proliferate and survive. The external signaling molecule that initiates the cascade may bind to a variety of extracellular receptors, including epidermal growth factor receptor or 5-HT_2_R, but MAPK-ERK signaling events converge on the kinases Raf, MEK, and ERK [100,102,103]. The importance of this signaling cascade in the JCPyV lifecycle is supported by the observation that the genetic knockdown of these kinases potently reduces JCPyV infection [67,69]. JCPyV-induced ERK phosphorylation occurs immediately upon virion internalization, peaks at two hours post internalization, and decreases until nine hours post internalization. Later ERK phosphorylation is also observed, which could facilitate late-stage events in the viral lifecycle, including replication [67,69,70]. Many of the transcription factors required for JCPyV infection, including the nuclear factor of activated T-cells (NFAT), are downstream of ERK, suggesting that ERK may be a critical mediator of virus replication in SVG-A cells [104]. 

Chemical inhibitors of the MAPK-ERK cascade, including U0126 (MEK1/2), PD98059 (MEK1), and sorafenib (c-Raf, receptor tyrosine kinase), potently reduce JCPyV infection in SVG-A cells by inhibiting ERK phosphorylation [67,101]. Other compounds, including genistein, target initial signaling events by inhibiting receptor tyrosine kinases [70]. Because other polyomaviruses, including SV40, do not require activation of the MAPK-ERK cascade to infect target cells, the effect of these MAPK-ERK antagonists seems specific to JCPyV [59,67,100]. 

In contrast to the virus-induced MAPK-ERK signaling events observed in SVG-A cells, JCPyV requires the phosphoinositide 3-kinase (PI3K)—protein kinase B (Akt)—molecular target of the rapamycin (mTOR) cascade to infect primary normal human astrocytes [59,67,69,70,97,100,101]. While the MAPK-ERK and PI3K-Akt-mTOR pathways show significant cross-inhibition and cross-activation, these cascades involve distinct kinases and phosphatases [105]. A distinct panel of signaling inhibitors is therefore effective in each target cell line. In primary normal human astrocytes, PI3K-Akt-mTOR inhibitors, including wortmannin (PI3K), MK2206 (Akt), and rapamycin (mTOR), reduce JCPyV infection, but MAPK-ERK inhibitors like U0126 lose antiviral activity [97]. However, MAPK-ERK inhibitors reduce JCPyV infection in normal human astrocytes when transformed with SV40 TAg, suggesting that the involvement of MAPK-ERK in SVG-A cells may be a consequence of immortalization. No clinical results have been reported with PML patients treated with signaling inhibitors [7]. The relative importance of each signaling pathway has not been explored in other cell types that may contribute to PML pathogenesis, including the choroid plexus epithelium and meninges [106]. As such, more research is needed to resolve these context-dependent complexities.

Signaling inhibitors with targets other than MAPK-ERK or PI3K-Akt-mTOR have also been reported to reduce JCPyV infection. The involvement of the calcium-sensitive transcription factor NFAT in JCPyV infection suggests that calcium signaling could play a role in the viral lifecycle, and topiramate, a blood–brain-barrier-permeable antagonist of calcium-mediated signaling, inhibits JCPyV infection [100,104,107]. Cyclosporine, the canonical inhibitor of calcineurin-NFAT signaling, also reduces JCPyV replication [108]. Additional inhibitors of calcium signaling also appear to possess anti-JCPyV activity via inhibition of calcium flux through the inositol triphosphate pathway, including U73122 (Phospholipase C), 2-APB (IP3R), and Xestospongin C (IP3R) [109]. Because calcium signaling is an extremely important physiological event that regulates diverse responses, including muscle contraction and neuronal conduction, targeting calcium efflux to reduce JCPyV infection could cause unintended deleterious side effects, especially within the CNS. The cyclin-dependent kinase inhibitor roscovitine also reduces JCPyV proliferation by inhibiting TAg phosphorylation in neuroblast-derived cell lines [110]. Despite the identification of these antivirals, the involvement of cyclin-dependent kinase-mediated and calcium-mediated signaling in JCPyV infection is not fully understood and requires further research.

### 4.5. Trafficking Inhibitors

JCPyV undergoes trafficking from the cellular periphery to the endoplasmic reticulum (ER) via microtubules and intermediate filaments [90,111,112]. This process, called retrograde transport, can be inhibited by the dihydroquinazolinones Retro-2^cycl^, Retro-2.1, and DHQZ36, which all prevent JCPyV colocalization with the ER [111,112]. These compounds are better tolerated in vitro than the canonical retrograde transport inhibitor brefeldin A, a macrocycle that reduces JCPyV infection by altering cellular recognition of vesicles destined for the ER [89]. Retro-2.1, a second-generation dihydroquinazolinone, reduces JCPyV infection at eightfold lower concentrations than the parent drug, Retro-2^cycl^, with minimal cytotoxic effects at therapeutic concentrations [112]. Preclinical studies evaluating the ability of dihydroquinazolinone to exert therapeutic activity within the CNS are lacking. 

Antimitotic compounds that alter cytoskeletal architecture also possess anti-JCPyV activity. Nocodazole and cytochalasin D, which inhibit microtubule and actin assembly, respectively, have been shown to inhibit JCPyV infection [90]. 

### 4.6. Uncoating Inhibitors

Once in the ER, resident isomerases catalyze changes in the viral capsid to reveal the JCPyV genome [59,111]. Viral uncoating may be calcium-dependent, as transfection by JC Pseudovirus, a novel virus-like particle used to study early events of the virus lifecycle, can be inhibited with thapsigargin, an inhibitor of ER-bound calcium channels [91]. 

### 4.7. Nuclear Transport Inhibitors

At multiple points in the virus lifecycle, JCPyV DNA or newly synthesized viral proteins traffic between the nucleus and the cytoplasm. These mechanisms are not unique to JCPyV and are shared by many host and viral proteins. The nuclear entry of polyomavirus capsid proteins and T-antigens appears to be mediated by importins, which direct proteins tagged with nuclear localization signals into the nucleus [113]. The canonical importin inhibitor MK-933 reduces BKPyV infection, but similar data have not been reported for JCPyV [114]. The nuclear export inhibitor verdinexor potently reduces JCPyV infection by disrupting transport of viral components out of the nucleus [115]. Verdinexor is licensed to treat canine lymphoma and shows antiviral activity against respiratory syncytial virus and influenza virus, underscoring the potential safety and efficacy of targeting nuclear export to reduce virus infection [116].

### 4.8. Large T-Antigen Inhibitors

JCPyV TAg contains an ATP-dependent helicase domain that unwinds viral DNA so various proteins involved in replication may interact with the NCCR. Other TAg domains also play an important role in the virus lifecycle via inhibition of host tumor suppressors and initiation of the cell cycle [117,118,119]. Because the TAg ATPase is the only enzyme encoded in the JCPyV genome, the possibility of developing virus-directed antivirals with limited off-target toxicity is extremely attractive. 

A high-throughput screen of blood–brain-barrier-permeable compounds identified LDN-0012754, a substituted indole with potent inhibitory activity of TAg ATPase [117]. Later studies investigated TAg inhibitors based on different heterocyclic cores, including carbonyl-containing dihydropyrimidinones and carbonylothioyl-containing dihydropyrimidinethiones. The most potent TAg inhibitor, AMT580-043, inhibits JCPyV infection at nearly fourfold smaller concentrations than the first-generation inhibitor SMAL. Interestingly, despite targeting a viral protein, AMT580-043 was more toxic in vitro than established anti-JCPyV compounds [120]. Some speculate that targeting TAg ATPase without affecting the other TAg domains may increase the risk of oncogenesis, as TAg is a potent oncogene [7]. Nevertheless, exploiting the structural differences between host and viral ATP-dependent helicases to develop specific antiviral agents warrants further compound optimization and preclinical investigation. 

### 4.9. Replication Inhibitors

Because JCPyV does not encode a polymerase, the virus relies on host machinery for replication. As such, many established antiviral and anticancer drugs have been evaluated as potential anti-JCPyV compounds. Due to their longer history, many of the metabolic steps required to activate these inhibitors have been extensively characterized. Replication inhibitors antagonize multiple aspects of JCPyV replication, including DNA elongation, nucleic acid synthesis, topoisomerase-mediated stabilization of the unwound DNA, and DNA repair mechanisms. 

Used to treat certain kinds of leukemia, a phosphorylated metabolite of the nucleoside analog cytarabine antagonizes viral replication by competitively inhibiting DNA polymerase [7]. Cytarabine potently inhibits JCPyV replication in a persistently infected cell line, and cytarabine treatment was first attempted in the early 1970s [121,122]. Since then, clinical trials have failed to identify any benefit associated with cytarabine therapy. Over 70% of patients in the cytarabine treatment group in one uncontrolled study of HIV-associated PML experienced disease progression and had increased JCPyV viral loads [123]. A later multicenter trial found no difference in the prognosis of HIV-associated PML patients when treated with either intravenous or intrathecal cytarabine, relative to treatment with HAART alone [124]. Importantly, cytarabine also causes significant neurological toxicity in up to 15% of patients. Intrathecal cytarabine is associated with irreversible spinal cord damage, and intravenous cytarabine is associated with peripheral neuropathy, seizures, and an acute cerebellar syndrome [125]. Due to the high toxicity and limited antiviral activity, cytarabine is not recommended for treatment of PML. 

Other pyrimidine nucleotide analogs show more promise as anti-JCPyV compounds. Cidofovir, an acyclic nucleotide analog, shows antiviral activity against other DNA viruses, including human cytomegalovirus, but fails to reduce JCPyV infection in vitro [7,122]. Multiple studies have found that cidofovir treatment is not associated with improved outcomes in HIV-associated PML, and that cidofovir in combination with HAART is not more effective than HAART alone [126,127]. This observation may be explained by failure of cidofovir to penetrate the CNS [7]. However, brincidofovir, a synthetic cidofovir prodrug, increases oral bioavailability and penetrates the blood–brain barrier in mice. Brincidofovir is a lipid conjugate of cidofovir, and the addition of this hydrophibic side group enhances the bioavailability of brincidofovir relative to cidifovir [128]. Now approved to treat smallpox infection, brincidofovir potently suppresses JCPyV replication and spread at nearly 100× smaller concentrations than required to achieve similar responses with cidofovir [129,130]. Oral formulations of brincidofovir have been prescribed as monotherapies or in combination with other anti-JCPyV agents, including mefloquine, risperidone and mirtazapine, with favorable clinical and virological results [131]. However, outcomes may depend on when brincidofovir treatment begins relative to PML onset, as one patient with drug-associated PML died after brincidofovir therapy, although treatment began 2 years after symptom onset [132]. While promising, larger trials of brincidofovir are needed to evaluate its efficacy as a therapeutic for PML. 

Leflunomide is a synthetic isoxazole with immunosuppressive effects that is prescribed to treat rheumatoid arthritis. Physiological cleavage of the isoxazole ring yields teriflunomide, the active metabolite with potent inhibitory activity against dihydroorotate dehydrogenase, an enzyme that catalyzes the rate-limiting step of uridine biosynthesis [133]. It is not surprising that some case reports associate leflunomide treatment with PML [134]. However, long-term studies of teriflunomide, which is prescribed to treat multiple sclerosis, fail to associate teriflunomide use with an increased risk of developing PML [135]. Interestingly, teriflunomide has been shown to inhibit JCPyV infection and spread in astrocytes and choroid plexus cells, and high blood teriflunomide concentrations are associated with reductions of BKPyV load in patients with BKPyV-associated nephropathy [133,136]. This apparent paradox may be resolved by the observation that coincubation of teriflunomide with uridine precursors downstream of dihydroorotate dehydrogenase fails to rescue JCPyV infection in vitro, suggesting that the antiviral mechanism of teriflunomide may derive from another interaction with an unidentified target [133]. In contrast, inhibition of BKPyV infection with teriflunomide is rescued by the addition of uridine, which may suggest that two polyomavirus-dependent antiviral mechanisms may exist [137]. While these data are promising, more work is needed to resolve these complexities.

JCPyV TAg recruits host topoisomerases to stabilizing unwinding of the JCPyV dsDNA. Many topoisomerase inhibitors have been screened for anti-JCPyV activity, including topotecan and β-lapachone. Both compounds are well-established chemotherapeutics and potently inhibit JCPyV replication, infection, and spread in neuroblastoma cells [138]. Results from a Phase II clinical trial showed that topotecan may reduce lesion size and prolong lifespan in HIV-associated PML, although further studies are needed to validate these results [139]. The ortho-naphthoquinone β-lapachone possesses modest anticancer activity when prescribed as a monotherapy. β-lapachone derivatives can cause significant anemia, but new formulations like ARQ791 seem to be associated with less dose-limiting toxicity [140]. Importantly, mouse and human studies show that both topotecan and β-lapachone can pass the blood–brain barrier [141,142]. Recent reports demonstrate that another blood–brain-barrier-permeable topoisomerase inhibitor, CPT11, has more potent anti-JCPyV activity than either topotecan or β-lapachone, even at concentrations as low as 1 μM [143,144]. These topoisomerase inhibitors therefore warrant further investigation as potential therapeutics for PML.

Viral replication also seems to involve host DNA repair mechanisms as compounds, including 3-aminobenzamide (3-AB) reduce JCPyV replication and spread by inhibiting PARP-1, an enzyme that mediates excision repair [145]. However, 3-AB is a representative inhibitor, and lead optimization is needed to identify more specific PARP-1 antagonists. 

### 4.10. Extracellular Vesicle Association Inhibitors

JCPyV association with EVs appears to involve multiple redundant packaging pathways, including secretory autophagy and exosome budding. In particular, the genetic knockdown of neutral sphingomyelinase 2 (nSMase2), an enzyme involved in sphingolipid metabolism and extracellular vesicle formation, reduces JCPyV spread and association with extracellular vesicles. Cambinol, a small molecule nSMase2 inhibitor, also reduces JCPyV spread over 9 days, suggesting that virus association with extracellular vesicles may be a viable therapeutic target to control PML progression in vivo [87].

### 4.11. Inhibitors of Unknown Mechanisms

Several small molecule antivirals have been discovered without any knowledge of their mechanism of action. One notable compound is artesunate, a semi-synthetic medication used to treat severe malaria. Artesunate is associated with a dose-dependent decrease in JCPyV replication and has been prescribed in combination with mirtazapine with no apparent clinical benefit [146,147]. Importantly, this result could be attributed to failure to penetrate the CNS, as no artesunate could be detected in the CNS two hours after intravenous administration in a clinical study of severe malaria [148]. A high-throughput screen identified ellagic acid and spiperone as compounds with anti-JCPyV activity. While the exact mechanism remains unknown, some hypothesize that the antioxidant effects of ellagic acid may be responsible for its antiviral activity. Surprisingly, although spiperone is a 5-HT_2_R inhibitor, this seems to be unrelated to its anti-JCPyV properties, as spiperone also inhibits infection by BKPyV and SV40, which do not require the serotonin receptor for entry [149]. 

**Table 1:** Small molecule inhibitors of JCPyV infection and spread in vitro. Antiviral compounds are separated by putative mechanism of action and year of identification.

Mechanism	Target	Compound Name	Year	Reference
Attachment inhibitor	LSTc binding pocket	AY4	2015	[65]
		Soluble LSTc	2013	[64]
Entry inhibitor	5-HT2R	Clozapine	2003	[72]
		Mirtazapine	2008	[71]
		Cyproheptadine		
		Risperidone		
		Ketanserin	2004	[66]
		Ritanserin		
		Mianserin		
		Volinsanserin		
		Metoclopramide		
	5-HT2R	Chlorpromazine	2000	[83]
	Clathrin-mediated endocytosis			
	Clathrin terminal domain	Pitstop2	2019	[68]
	Dynamin	Dynole 34-2		
	Macropinocytotis	EIPA	2020	[85]
Endosomal acidification inhibitor	Unknown	Mefloquine	2009	[92]
	Na+/H+ ATPase	Monensin	2013	[91]
	Vacuolar H+ ATPase	Bafilomycin A1		
Uncoating inhibitor	SERCA	Thapsigargin		
Receptor tyrosine kinase inhibitor	Receptor tyrosine kinase	Genistein	2004	[70]
MAPK-ERK signaling inhibitor	c-Raf	GW-5074	2023	[150]
		Sorafenib	2019	[101]
	MEK1/2	U0126	2018	[67]
		PD98059		
PI3K-AKT-mTOR signaling inhibitor	AKT	MK2206	2021	[97]
	PI3K	Wortmannin		
	mTOR	PP242		
		Rapamycin		
Ca2+ signaling	Calcineurin	Cyclosporine	2012	[108]
	L-type Ca2+ channels	Topiramate	2020	[107]
	Phospholipase C	U73122	2018	[109]
	IP3R	2-APB		
		Xestospongin C		
Cyclin-dependent kinase inhibitor	CDK2, CDK7, CDK9	Roscovitine	2008	[110]
Cytoskeleton disruptors	β-tubulin	Nocodazole	2003	[90]
	F-actin	Cytochalasin D		
Retrograde transport inhibitors	ASNA1	Retro-2cycl	2013	[111]
		DHQZ36		
		Retro-2.1	2020	[112]
	ADP-ribosylation factor	Brefeldin A	2006	[89]
TAg inhibitor	TAg ATPase	SMAL	2016	[120]
		AMT580-043		
		LDN-0012754	2014	[117]
Nucleoside analog	DNA Polymerase	Cytarabine	1974	[121,122]
Nucleotide analog	DNA Polymerase	Cidofovir		
		Brincidofovir	2010	[129,130,132]
Pyrimidine synthesis inhibitor	Dihydroorate dehydrogenase	Teriflunomide	2006	[133]
Topoisomerase inhibitor	Topoisomerase	β-lapachone	2016	[138]
		Topotecan		
		CPT11	2017	[143]
DNA repair inhibitor	PARP-1	3-AB	2013	[145]
Nuclear export inhibitor	SINE	Verdinexor	2018	[115]
Extracellular vesicle association inhibitor	nSMase2	Cambinol	2022	[87]
Unknown	Unknown	Artesunate	2014	[146]
		Ellagic acid	2009	[149]
		Spiperone		

## 5. GW-5074: A Novel JCPyV Inhibitor That Antagonizes Signaling Events

A major research focus in our laboratory is the development of new anti-JCPyV compounds. This begins by understanding the viral lifecycle, which can inform new targets for host-directed antivirals. Recent research into the mechanisms by which viruses co-opt host signaling networks to enter the cell, replicate their genetic material, and evade immune detection has generated significant interest in targeting signaling cascades to inhibit viral infection. Signaling inhibitors are a promising class of compounds because many have clinical use as anticancer therapeutics, so extensive preclinical studies have already established pharmacodynamic and safety profiles. For instance, the FDA-approved anticancer therapy sorafenib has been shown to reduce JCPyV infection by inhibiting c-Raf, a central kinase in the MAPK-ERK cascade [69]. However, sorafenib fails to cross into the CNS, and therefore is unlikely to be an effective therapy for PML [151]. Because c-Raf plays an important role in JCPyV infection, and the safety of targeting c-Raf has already been established with cancer therapies like sorafenib, we sought to characterize blood–brain-barrier-permeable c-Raf inhibitors as novel anti-JCPyV agents.

We selected GW-5074 (3-(3,5-dibromo-4-hydroxybenzylidene)-5-iodoindolin-2-one) to screen for antiviral activity. Initially synthesized in 2000 by Lackey et al., GW-5074 shows potent and specific inhibitory activity of c-Raf [152]. We were drawn to this compound because of its oxindole core. Oxindole (2-indolone) has been used as a nucleus to synthesize many structurally diverse pharmacological compounds with different biological activities, including anticancer, antibacterial, antiviral, and antifungal effects (Table 2) [153,154,155]. Two oxindole-based compounds, nintedanib and sunitinib, antagonize signaling events and are clinically-used cancer therapeutics [156,157,158,159,160,161,162,163,164]. Ropinirole and ziprasidone are approved to treat Parkinson’s Disease and schizophrenia, respectively, which underscores the potential of oxindole-based drugs to pass the blood–brain barrier and exert therapeutic effects within the CNS [165,166,167]. Oxindole-based antivirals also reduce HIV-1 and parvovirus B19 infection [168,169].

Pretreating transformed SVG-A glial cells and primary normal human astrocytes (NHA) with non-toxic concentrations of GW-5074 potently reduced initial JCPyV infection, as evaluated by indirect immunofluorescent quantification of viral proteins after one round of productive infection. The 50% inhibitory concentration (IC_50_) associated with GW-5074 treatment was 6.8 μM and 0.84 μM in SVG-A and NHA cells, respectively. GW-5074 was well-tolerated by both cell lines, as the 50% cytotoxic concentration (CC_50_) associated with GW-5074 treatment was greater than 100 μM and 20 μM in SVG-A and NHA cells, respectively. Using these values, we calculated the minimum 50% selectivity index (SI_50_), defined as the ratio between the CC_50_ and IC_50_, associated with GW-5074 to be 11.8 in SVG-A cells and 20.2 in NHAs [150]. SI_50_ values are important metrics in drug discovery, and an SI_50_ greater than 10 is commonly considered to be an important starting point in drug development [7]. The SI_50_ associated with GW-5074 treatment is larger than the SI_50_ values associated with established compounds with anti-JCPyV activity, including chlorpromazine (SI_50_ = 2.0), mefloquine (SI_50_ = 4.0), and Retro-2^cycl^ (SI_50_ = 7.4) [7]. Importantly, GW-5074 also inhibited long-term cell-to-cell spread of JCPyV when introduced after establishment of a productive infection. This result suggests that GW-5074 could be used to control PML in vivo, as most patients support uncontrolled virus replication before symptom onset [150]. 

**Figure 4:**
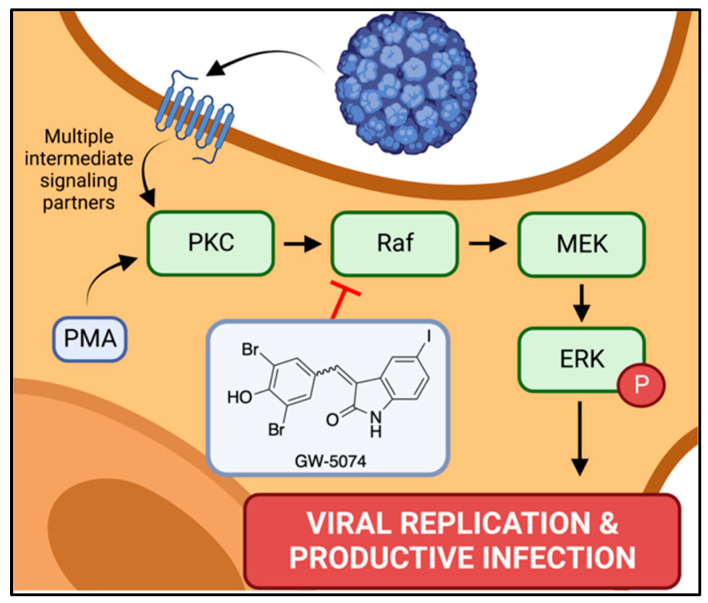
Proposed anti-JCPyV mechanism of GW-5074. GW-5074 is known to antagonize MAPK-ERK signaling by inhibiting c-Raf. This mechanism may underpin its anti-JCPyV activity. Created with BioRender.com.

Because GW-5074 is known to inhibit c-Raf, we suspected that the antiviral activity of GW-5074 was associated with its antagonistic effects on MAPK-ERK signaling. We observed that GW-5074 inhibited endogenous ERK phosphorylation, and that ERK activity could not be restored upon coincubation with the Protein Kinase C (PKC) agonist phorbol 12-myristate 13-acetate (PMA). PKC directly activates c-Raf, so these results are consistent with the known signaling antagonism of GW-5074. Similar results were obtained in the context of virus infection, as JCPyV infection could not be rescued by cotreating SVG-A cells with PMA and GW-5074 [150]. Taken together, these data support the hypothesis that GW-5074 disrupts MAPK-ERK signaling events needed to establish a productive JCPyV infection (Figure 4).

However, our experiments also suggest that GW-5074 may have another mechanism underpinning its antiviral activity. MAPK-ERK inhibitors fail to reduce JCPyV infection in NHAs because JCPyV seems to co-opt the PI3K-Akt-mTOR cascade in these primary cells [97]. Although crosstalk has been characterized between molecular players in the PI3K-Akt-mTOR and MAPK-ERK pathways, we were surprised to observe that the antiviral activity of GW-5074 was more dramatic in NHAs [105]. Additionally, SV40 is not sensitive to MAPK-ERK inhibitors, but GW-5074 reduced SV40 infection in SVG-A cells [67]. These potentially conflicting results may be explained by observations that GW-5074 disrupts nuclear import of viral proteins by altering the importin α/$\beta_{1}$ heterodimer [170]. Although small molecule inhibitors of nuclear import, including MK-933, are not known to reduce JCPyV infection, our experiments do not rule out the possibility that disrupting nuclear transport may play a role in the antiviral activity of GW-5074, especially in contexts where MAPK-ERK signaling does not mediate JCPyV infection.

Ultimately, our laboratory has characterized GW-5074 as a novel antiviral agent that potently reduces JCPyV infection in spread in primary and transformed cell lines. Phase I clinical trials evaluating whether combination therapies of sorafenib and GW-5074 can inhibit tumor growth show that GW-5074 is largely well-tolerated, but poor water solubility of this highly lipophilic compound reduces its bioavailability. However, novel oral formulations, including MG010, an ionic salt formed by combining an ammonium cation with the phenoxide anion of GW-5074, increase drug solubility in water almost 20-fold relative to unsalted GW-5074 [171]. Further experiments in our laboratory are focused on evaluating whether GW-5074 can exert therapeutic activity within the CNS by using in vitro and in silico models of the barriers that restrict access to the CNS.

## 6. Current Obstacles in PML Drug Development

Extensive preclinical research has identified numerous compounds that inhibit JCPyV infection in vitro, but none have become approved therapeutics with clear clinical benefits. The obstacles hindering drug discovery can be attributed to three main factors, including the relative rarity of developing PML, the absence of a PML animal model, and the challenges associated with designing compounds that exert therapeutic activity within the CNS. 

### 6.1. Obstacle 1: PML Is a Rare Disease 

The incidence of PML is around 1 in 200,000, and just 4000 people are estimated to be diagnosed with PML per year in the United States and Europe [40]. PML also progresses rapidly, which complicates the organization of the randomized clinical trials needed to evaluate the efficacy of novel antivirals [172]. In the 70-year history of PML, 24 clinical trials associated with PML have been recorded. Only seven tested antiviral compounds: four clinical trials evaluated small molecules (mefloquine, cytarbine, topotecan, cidofovir, and zidovudine) while two clinical trials examined immunotherapies (pembrolizumab, NT-I7), and one clinical trial evaluated a combination therapy of a small molecule and an immunotherapy (zidovudine and α interferon) (Table 3). These clinical trials were mostly small, with the largest including 90 patients. At the time of this writing, one clinical trial evaluating the use of NT-I7, a recombinant human interleukin 7, was still ongoing (ID: NCT04781309). Ultimately, large, randomized clinical trials are needed to develop new treatments for PML, but patient recruitment is challenging due to the low incidence and rapid progression of the disease.

### 6.2. Obstacle 2: No Reliable Animal Model for PML Exists

Like other polyomaviruses, JCPyV infection is highly species-specific. Studies with mouse polyomavirus (MPyV) and BKPyV suggest that this narrow tropism could arise from the interaction of host factors, particularly host DNA polymerase, with the viral origin of replication [173,174]. Intracerebral inoculation of non-human primates, hamsters, and transgenic mice with JCPyV results in non-productive viral infection, characterized by overexpression of the early gene products, including TAg. Instead of inducing a PML-type demyelinating phenotype, high TAg expression promotes integration of the viral DNA into the host genome, which favors tumor formation [175,176,177,178].

Despite these obstacles, significant progress has been made towards developing an animal model for PML. One notable approach is repurposing MPyV as a JCPyV-type agent in mice. These two polyomaviruses share many virological and immunological similarities, including induction of disease following immunosuppression, persistence in the renal–urinary system, and infection of oligodendrocytes, choroid plexus epithelial cells, and meningeal cells [179,180,181,182,183]. MPyV and JCPyV also seem to have similar interactions with the immune system, particularly CD8+ T-cells [180,181,182,183,184]. However, unlike the multifocal white matter lesions characteristic of PML, MPyV neuropathology is largely defined by generalized encephalopathy with demyelination [183]. 

The absence of a reliable animal model for PML has severely inhibited the development of an effective therapeutic. Except for Retro-2.1, which has been shown to reduce MPyV viral burden in immunosuppressed mice, most anti-JCPyV compounds are evaluated in patients following tissue culture studies because there is no intermediary animal model [185]. Escalating directly to patient administration skips the preclinical studies needed to optimize antiviral activity, including studies of drug metabolism, in vivo toxicity, and CNS penetration.

### 6.3. Obstacle 3: Few Compounds Accumulate in the CNS

Because JCPyV is a neurotropic virus, an effective antiviral agent will need to pass into the CNS. While intrathecal antiviral administration maximizes compound bioavailability within the CNS, there are many disadvantages associated with this route, including (1) the surgical challenges involved in drug administration and (2) patient inconvenience and pain [186,187]. As such, the ideal anti-JCPyV therapeutic would be delivered via an oral or intravenous route. However, only compounds with a narrow range of drug-like physiochemical properties will effectively diffuse across the barriers that restrict access to the CNS. While the usefulness of different qualitative rules is controversial, molecular size (MW), total polar surface area (TPSA), the number of hydrogen bond donors (HBD), serum binding, and lipophilicity (cLogP) appear predictive of CNS accumulation. CNS-targeting drugs are typically small (MW ≤ 450 Da, TPSA < 60 Å^2^), non-polar (HBD ≤ 2, cLogP < 5), and bind poorly to serum proteins (K_d_ albumin < 10 μM) [188,189,190,191]. P-glycoproteins (P-gp), a family of efflux transporters highly expressed in the blood–brain barrier, significantly restrict small molecule access to the CNS; most CNS-targeting drugs are not P-gp substrates [191]. Additionally, CNS-targeting compounds should be stable in the plasma and resistant to metabolism by hepatic Cytochrome P450s, which catalyze oxidations of foreign compounds, increase compound polarity, and may transform a drug into a metabolite with no therapeutic activity [192].

Preclinical studies of anti-JCPyV agents should attempt to evaluate whether lead compounds will accumulate in the CNS. While in vivo pharmacodynamic studies are important, these experiments are costly and require ethical considerations. Multiple accessible in vitro systems exist that model drug diffusion across the barriers that restrict access to the CNS [193]. Additionally, in silico approaches, including the SwissADME Web Tool, can also help researchers evaluate lead compounds before performing expensive in vitro or in vivo experiments [194].

## 7. Conclusions

The current panel of anti-JCPyV compounds includes attachment inhibitors, entry inhibitors, transport inhibitors, TAg helicase inhibitors, replication inhibitors, and extracellular vesicle inhibitors. Because JCPyV is a relatively simple virus, most anti-JCPyV compounds antagonize a host process required for virus infection rather than a viral component. Co-opting cancer therapeutics to antagonize virus-induced cell signaling events may be a promising new approach to antiviral drug development, as the safety and pharmacokinetic profiles of these approved inhibitors are already well-characterized. One novel signaling inhibitor, the oxindole GW-5074, potently reduces JCPyV infection and spread in primary and immortalized cells. Despite identification of these druggable targets, in vitro inhibitors of virus infection and spread have largely failed to treat or prevent PML in vivo. Researchers must continue to explore new treatment strategies to overcome the virological and physiological obstacles that have hindered previous drug development efforts in order to develop viable therapies for PML. 

## Figures and Tables

**Table 2:** Structures of approved oxindole-based compounds. The oxindole core is highlighted in red.

Compound Structure	Commercial Name	Disease Target(s)	Year Licensed	Reference
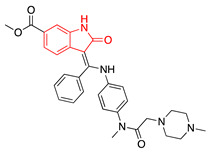	Nintedanib	Idiopathic pulmonary fibrosis	2014	[156,157,158,159,160]
		Non-small-cell lung cancer		
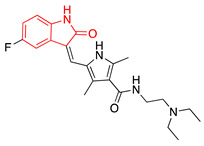	Sunitinib	Renal cell carcinoma	2006	[161,162,163,164]
		Gastrointestinal stromal tumor		
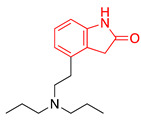	Ropinirole	Parkinson’s disease	1997	[165,166]
		Restless leg syndrome		
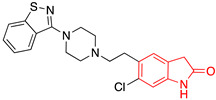	Ziprasidone	Schizophrenia	2001	[167]
		Bipolar disorder		

**Table 3:** Clinical trials for PML therapeutics as of 2023. From clinicaltrials.gov.

Identifier	Phase	Drug(s)	Drug Class	Size	Status
NCT00746941	II	Mefloquine	Small Molecule	37	Terminated
NCT00001048	II	Cytarabine	Small Molecule	90	Terminated
NCT00002395	II	Topotecan	Small Molecule	54	Completed
NCT04091932	II	Pembrolizumab	Antibody	10	Unknown
NCT04781309	I	NT-I7	Recombinant interleukin	12	Recruiting
NCT00002270	N/A	α interferon, zidovudine	Combination	Unknown	Unknown
NCT00000945	N/A	Cidofovir	Small Molecule	24	Completed

## Data Availability

No new data are presented in this study. All data can be accessed at the appropriate reference.
